# Fatal Case of Diphtheria and Risk for Reemergence, Singapore

**DOI:** 10.3201/eid2411.180198

**Published:** 2018-11

**Authors:** Yingqi Lai, Parthasarathy Purnima, Marc Ho, Michelle Ang, Rama N. Deepak, Ka Lip Chew, Shawn Vasoo, Dimatatac F. Capulong, Vernon Lee

**Affiliations:** Ministry of Health, Singapore (Y. Lai, M. Ho, M. Ang, V. Lee);; Khoo Teck Puat Hospital, Singapore (P. Purnima, R.N. Deepak);; National Public Health Laboratory, Singapore (M. Ang);; National University Hospital, Singapore (K.L. Chew);; Tan Tock Seng Hospital, Singapore (S. Vasoo, D.F. Capulong)

**Keywords:** diphtheria, Corynebacterium diphtheriae, bacteria, toxigenic, public health, vaccines, contact tracing, transients and migrants, reemergence, Singapore

## Abstract

We report a fatal autochthonous diphtheria case in a migrant worker in Singapore. This case highlights the risk for individual cases in undervaccinated subpopulations, despite high vaccination coverage in the general population. Prompt implementation of public health measures and maintaining immunization coverage are critical to prevent reemergence of diphtheria.

Diphtheria is an acute respiratory disease caused by infection with *Corynebacterium diphtheriae*. Case-fatality rates for respiratory diphtheria reached 50% in epidemics in the 1880s and remain ≈5%–10% even after use of antitoxin. Virulence is mediated by production of toxin by the *tox* gene of β-corynebacteriophage (*1*).

Global incidence of diphtheria decreased by >90% during 1980–2016 after initiation of the World Health Organization Expanded Programme on Immunization in 1974 (*2*). This decrease was also reflected by the shifting age distribution from children to adolescents and adults (*3*). Although the World Health Organization South-East Asia Region was estimated to contain 55%–99% of all reported diphtheria cases during 2011–2015, diphtheria cases have been reported in industrialized countries (*2**,**4**–**7*).

Singapore is a densely populated (8,000 persons/km^2^) city-state in Southeast Asia and a trade and travel hub. It last had an autochthonous case of toxigenic *C. diphtheriae* in 1992. This low incidence has been achieved by ensuring high population immunity through mandatory vaccination since 1962. A primary course of diphtheria, tetanus, and acellular pertussis vaccine is given to infants at 3, 4, and 5 months of age; booster vaccines are given at 18 months and 10–11 years of age. Immunization coverage for 2-year-olds has been >95% for the past 14 years (*8*).

On August 3, 2017, the Ministry of Health Singapore was notified of a diphtheria case in a person who had no recent travel overseas or known contact with an infected person. We describe the public health measures implemented to prevent further spread, and discuss risks for diphtheria reemergence in countries with high vaccination coverage, particularly those with insufficiently vaccinated subpopulations.

## The Study

A 23 year-old man from Bangladesh who had been a construction worker in Singapore for the previous 10 months had fever, sore throat, and neck pain develop on July 30, 2017. He visited a primary care clinic on July 31 and was given symptomatic treatment. 

His symptoms worsened, and he came to an emergency department because of odynophagia and hemoptysis on August 1. He was hypoxic and needed ventilation through a tracheostomy. Throat examination and subsequent intraoperative findings showed extensive soft palate edema obscuring the uvula and tonsils; purulent secretions obstructing the airway; and pseudomembranous mucosa over bilateral, necrotic-looking tonsils, the base of the tongue, and the larynx. Computed tomography showed extensive soft tissue edema causing near-complete airway narrowing from the choana to supraglottis and multiple enlarged cervical lymph nodes.

We made a clinical diagnosis of respiratory diphtheria. The patient was isolated immediately and given diphtheria antitoxin and intravenous erythromycin. However, his condition deteriorated rapidly, and he died of respiratory obstruction 48 hours after admission.

We obtained a laboratory diagnosis through parallel culture of tonsillar and pharyngeal tissue on trypticase soy agar containing 5% sheep blood, chocolate agar, and MacConkey agar. All cultures were incubated at 37°C in an atmosphere of 5% CO_2_ in a primary laboratory. Culture of laryngeal and nasal discharge was performed in the referral laboratory on Tinsdale Columbia CNA agar (Becton Dickinson, Sparks, MD, USA) containing 5% sheep blood and sheep blood agar. Bacterial colonies grew in all cultures; gram-positive rods were identified as *C. diphtheriae* by using the API Coryne System (BioMérieux, Marcy l’Etoile, France) and Bruker MALDI Biotyper (Bruker Daltonics, Bremen, Germany). We detected toxin A and B subunits of the *C. diphtheriae* toxin (*tox*) gene in the tonsil isolate by using an adapted PCR (*9*).

**Figure 1:**
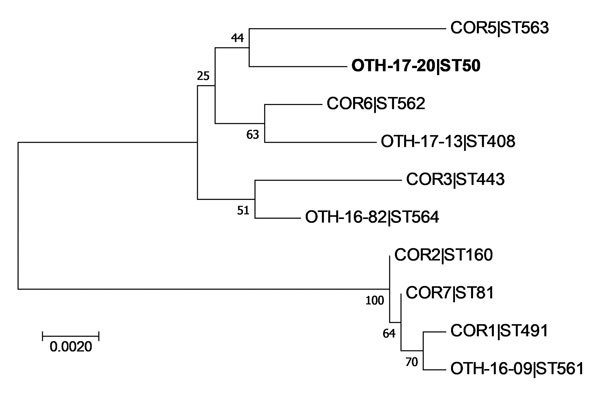
Phylogenetic analysis of *Corynebacterium diphtheriae* isolate from a 23-year-old man who died from diphtheria (OTH-17-20; bold) and 9 other isolates collected from hospitals in Singapore during 2013–2017. The tree was constructed by using 7 concatenated housekeeping gene sequences corresponding to the *C. diphtheriae* multilocus sequence typing scheme (https://pubmlst.org/cdiphtheriae/). Sequences were extracted from whole-genome sequences of each isolate. Concatenated sequences were aligned by using ClustalW (http://www.clustal.org/). Phylogeny was inferred by using the maximum-likelihood method, neighbor-joining algorithm based on the Jukes-Cantor model, and MEGA7 software (*10*). There were 2,544 positions in the final dataset. Numbers next to branches show bootstrap values calculated by using 1,000 reiterations. Scale bar indicates nucleotide substitutions per site. ST, sequence type.

**Figure 2:**
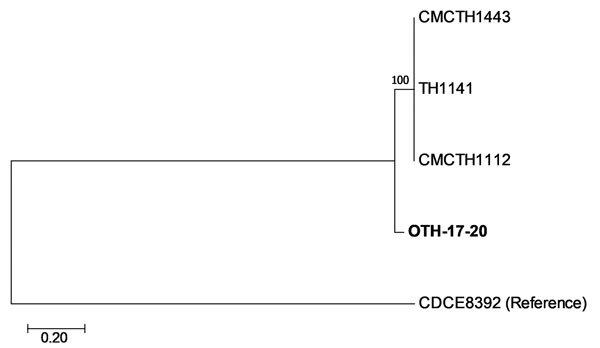
Core-genome single-nucleotide polymorphism (SNP) phylogeny of *Corynebacterium diphtheriae* isolate from a 23-year-old man who died from diphtheria (OTH-17-20; bold) and 4 publicly available ST50 genomes (TH1141, GenBank accession no. GCA_001723455.1; CMCTH1443, accession no. GCA_001981275.1; CMCTH1112, accession no. GCA_001981275.1; and CDCE8392, accession no. GCA_000255215.1) of isolates collected from hospitals in Singapore during 2013–2017. Phylogeny was deduced by alignment with Parsnp (http://harvest.readthedocs.io/en/latest/content/parsnp.html), a rapid core-genome multialignment tool for genome assemblies. Extracted SNPs were concatenated, and phylogeny was inferred as described in Figure 1. There were 26,785 positions in the final dataset. Numbers next to branches show bootstrap values calculated by using 1,000 reiterations. Scale bar indicates nucleotide substitutions per site. ST, sequence type.

The patient isolate (OTH-17-20) was confirmed as toxigenic *C. diphtheriae* biovar mitis. In silico multilocus sequence typing (MLST) showed that OTH-17-20 belonged to sequence type (ST) 50, which was unrelated to the STs of nontoxigenic isolates obtained from public hospitals in Singapore during 2013–2017 (BioProject PRJNA445775) (Figure 1). When we compared OTH-17-20 with 4 other available ST50 genomes in GenBank by core-genome, single-nucleotide polymorphism analysis, we found that this isolate was more related to the cluster of 3 ST50 genomes from India than to 1 isolate from Germany (Figure 2; Technical Appendix.

Movement of the patient was confined to his dormitory room and worksite. Because the moribund patient could not communicate, we conducted interviews with his colleagues, employer, and dormitory roommates to ascertain possible exposure(s). These interviews included identifying close contacts, who were defined as persons with similar proximity and duration of exposure as household contacts who were directly exposed to large-particle droplets or secretions from the patient (*11*).

Adopting a precautionary approach, we identified 3 groups of contacts. The first group was 11 persons who lived in the same dormitory room. The second group was 37 colleagues who worked together or shared transportation to the workplace. The third group was 72 healthcare workers who provided direct care to the patient without adequate personal protective equipment.

Among the second group, 3 persons had sore throats develop; they were immediately placed in isolation in the hospital. Nasopharyngeal and throat swab specimens obtained from these 3 patients before they received antimicrobial drugs were negative for *C. diphtheriae*. All 3 patients recovered without complications. 

Dormitory and workplace contacts were of various nationalities (age range 22–39 years). All contacts were given 7 days of oral erythromycin or clarithromycin and a diphtheria toxoid booster vaccination if they had not received this vaccination in the previous 5 years or if vaccination history was unknown. Most dormitory and workplace contacts could not recall their vaccination history or did not have vaccination records.

To determine possible sources of exposure or onward transmission, we obtained nasopharyngeal and throat swab specimens before administration of antimicrobial drugs on August 4 from dormitory and workplace contacts for concurrent culture on selective media and testing by PCR. All swab specimens were negative for *C. diphtheriae*, and no secondary cases were detected at the end of 2 incubation periods.

## Conclusions

We report potential reemergence of locally transmitted toxigenic diphtheria in Singapore. As with cases in other industrialized countries, investigations could not identify a source of infection (*4**,**7*). Asymptomatic carriers remained a possibility because studies have reported bacterial carriage in vaccinated persons and nontoxigenic strains that underwent phage conversion (*5**,**6**,**12**–**15*). Because the patient had an unknown vaccination history, missing childhood vaccinations might have put him at risk for infection.

Such risks from severe vaccine-preventable diseases are present because of movement of unvaccinated or inadequately vaccinated persons. For migrants, these risks are compounded by population density and working conditions. Singapore hosts workers from countries such as India, Bangladesh, and Myanmar, to which diphtheria is endemic (*2*). Although these countries have now achieved high immunization coverage, levels were much lower decades ago when current migrant workers were children. In Bangladesh, where the patient originated from, 97% of infants received a primary course of diphtheria vaccine in 2016, compared with 84% in 1994 when the patient was an infant (*2*). Seroprotection has been shown to wane over time after a primary infant series (*1*). Therefore, booster doses during adolescence and adulthood are needed to maintain immunity.

This fatal case is a reminder that unvaccinated persons among specific subpopulations remain vulnerable to severe vaccine-preventable diseases, such as diphtheria. Clinicians and public health practitioners should remember that primary prevention through up-to-date vaccinations are necessary not only for children but also for adults who do not have appropriate coverage. In this regard, Singapore implemented the National Adult Immunization Schedule in 2017 to increase vaccination uptake.

Similar to other diseases with widespread childhood vaccine coverage, diphtheria has been mostly forgotten among healthcare professionals in industrialized countries. Clinical suspicion and diagnosis of this case was instrumental for rapid implementation of control measures. Clinicians, microbiologists, and public health specialists should be reminded of the risk for reemerging diphtheria.

Technical Appendix Additional information on fatal case of diphtheria and risk for reemergence, Singapore.
