# Supplementary material for: Fatal Case of Diphtheria and Risk for Reemergence, Singapore
Source: Emerg Infect Dis. 2018 Nov;24(11):2084–6. doi: 10.3201/eid2411.180198 (PMC6199979; doi:10.3201/eid2411.180198)
Supplement: Technical Appendix — Additional information on fatal case of diphtheria and risk for reemergence, Singapore. [file 18-0198-Techapp-s1.pdf]

Article DOI: https://doi.org/10.3201/eid2411.180198

# Fatal Case of Diphtheria and Risk for Reemergence, Singapore

## Technical Appendix

### Whole-Genome Sequencing Analysis

For preparation of whole-genome sequence libraries, purified DNA was quantified and processed by using the Nextera XT Library Preparation Kit (Illumina, Inc., San Diego, CA, USA) for generation of sequencing libraries as per the manufacturer's instructions. Multiplex paired-end libraries (2 × 250–300 bp) were sequenced on an Illumina Miseq instrument and the Miseq v3 protocol. In silico multilocus sequence typing (MLST) based on the *Corynebacterium diphtheriae* MLST scheme (https://pubmlst.org/cdiphtheriae/info/protocol.shtml) was inferred from the whole-genome sequence by using SRST2 (*1*). MLST sequences were obtained by read mapping genome data to a database of reference allele sequences (downloaded from PubMLST) to extract a consensus sequence for each allele for each isolate. Multiple alignment and phylogenetic analysis was performed by using MEGA7 (*2*). The mlst tool (https://github.com/tseemann/mlst) was used to deduce the sequence type (ST) for 199 publicly available *C. diphtheriae* genomes downloaded from Genbank, of which 4 were found to belong to ST50. Of the 4 ST50 publicly available genomes, 3 were submitted from India (GCA_001723455.1, GCA_ 001935895.1, and GCA_001981275.1) and 1 from Germany (GCA_000255215.1). The genome submitted from Germany was originally obtained from the Centers for Disease Control and Prevention (*3*). Rapid core genome single-nucleotide polymorphism analyses were performed by using parsnp (*4*).

### References

1. Inouye M, Dashnow H, Raven LA, Schultz MB, Pope BJ, Tomita T, et al. SRST2: rapid genomic surveillance for public health and hospital microbiology labs. Genome Med. 2014;6:90. PubMed http://dx.doi.org/10.1186/s13073-014-0090-6

2. Kumar S, Stecher G, Tamura K. MEGA7: Molecular Evolutionary Genetics Analysis Version 7.0 for bigger datasets. Mol Biol Evol. 2016;33:1870–4. PubMed http://dx.doi.org/10.1093/molbev/msw054

3. Trost E, Blom J, Soares SC, Huang IH, Al-Dilaimi A, Schröder J, et al. Pangenomic study of *Corynebacterium diphtheriae* that provides insights into the genomic diversity of pathogenic isolates from cases of classical diphtheria, endocarditis, and pneumonia. J Bacteriol. 2012;194:3199–215. PubMed http://dx.doi.org/10.1128/JB.00183-12

4. Treangen TJ, Ondov BD, Koren S, Phillippy AM. The Harvest suite for rapid core-genome alignment and visualization of thousands of intraspecific microbial genomes. Genome Biol. 2014;15:524. PubMed http://dx.doi.org/10.1186/s13059-014-0524-x
